# Quality of Life Framework for Personalised Ageing: A Systematic Review of ICT Solutions

**DOI:** 10.3390/ijerph17082940

**Published:** 2020-04-24

**Authors:** Sabina Baraković, Jasmina Baraković Husić, Joost van Hoof, Ondrej Krejcar, Petra Maresova, Zahid Akhtar, Francisco Jose Melero

**Affiliations:** 1 Faculty of Transport and Communications, University of Sarajevo, Zmaja od Bosne 8, 71000 Sarajevo, Bosnia and Herzegovina; 2 American University in Bosnia and Herzegovina, Zmaja od Bosne 8, 71000 Sarajevo, Bosnia and Herzegovina; 3 Little Mama Labs, Gradačačka 29, 71000 Sarajevo, Bosnia and Herzegovina; jasmina_barakovic@yahoo.com; 4 Faculty of Electrical Engineering, University of Sarajevo, Zmaja od Bosne bb, 71000 Sarajevo, Bosnia and Herzegovina; 5 Chair of Urban Ageing, Faculty of Social Work & Education, The Hague University of Applied Sciences, Johanna Westerdijkplein 75, 2521 EN Den Haag, The Netherlands; j.vanhoof@hhs.nl; 6 Department of Spatial Economy, Faculty of Environmental Engineering and Geodesy, Wrocław University of Environmental and Life Sciences, ul. Grunwaldzka 55, 50-357 Wrocław, Poland; 7 Faculty of Informatics and Management, University of Hradec Kralove, Rokitanskeho 62, 500 03 Hradec Kralove, Czech Republic; ondrej.krejcar@uhk.cz (O.K.); petra.maresova@uhk.cz (P.M.); 8 Department of Computer Science, University of Memphis, 234 Dunn Hall, Memphis, TN 38152, USA; zahid.eltc@gmail.com; 9 Technical Research Centre of Furniture and Wood of the Region of Murcia, C/Perales S/N, 30510 Yecla, Spain; fj.melero@cetem.es; 10 Telecommunication Networks Engineering Group, Technical University of Cartagena, 30202 Cartagena, Spain

**Keywords:** ICT, older adults, patent, personalised ageing, quality of life, review, smart ageing

## Abstract

Given the growing number of older people, society as a whole should ideally provide a higher quality of life (QoL) for its ageing citizens through the concept of personalised ageing. Information and communication technologies (ICT) are subject to constant and rapid development, and can contribute to the goal of an improved QoL for older adults. In order to utilise future ICT solutions as a part of an age-friendly smart environment that helps achieve personalised ageing with an increased QoL, one must first determine whether the existing ICT solutions are satisfying the needs of older people. In order to accomplish that, this study contributes in three ways. First, it proposes a framework for the QoL of older adults, in order to provide a systematic review of the state-of-the-art literature and patents in this field. The second contribution is the finding that selected ICT solutions covered by articles and patents are intended for older adults and are validated by them. The third contribution of the study are the six recommendations that are derived from the review of the literature and the patents which would help move the agenda concerning the QoL of older people and personalised ageing with the use of ICT solutions forward.

## 1. Introduction

According to the latest statistics from the World Health Organization (WHO) and the United Nations (UN) [1,2], the number of people 60 years and over will increase from 900 million to 2 billion by 2050 [1]. Furthermore, it is shown that the population is ageing quicker than it used to, especially in developing societies compared to developed ones [2]. Older people are estimated to make up 22% of the entire world population. The ageing of society is not as straightforward as it seems. The usual approach to ageing is that of calendar years, which is an objective indicator of age, and which completely neglects biological, subjective, and sociological ageing, as well as many other aspects of it. What the world needs is a more nuanced approach that reflects the multidimensional aspects of ageing. Therefore, the societies that address ageing in a smart and multidimensional longevity-centred way will have vast economic and social opportunities before them. 

Older adults require specific technological solutions that open a new market, which could stimulate the industry [3]. The creation of new products and services for older adults will, in turn, lead to new jobs and new companies in many innovative disciplines, such as electrical and software engineering, robotics, artificial intelligence, and so on. The economic potential of older adults should serve to change the ageing perception. Although ageing is usually connected with the loss of capabilities, older adults should stay active, since the professional experience and spending power of older adults could contribute to economic growth and development. Even more, in order to ensure sustainable society development in a future world where an average age is expected to be quite high, society as a whole must provide a high Quality of Life (QoL) for its ageing citizens [4]. In order to achieve a high QoL while ageing, one needs to have a personalised and individual approach regarding all its components. Personalised ageing refers to the fact that successful ageing is always connected to a person’s unicity [5]. Achieving personalised ageing is a very challenging and time-consuming task, which requires reshaping our current social concepts and behaviours and producing effective models and frameworks. 

Inevitably, intertwined personalised ageing and QoL as multidimensional concepts need to be treated differently in the ever-omnipresent digital environment, while at the same time, smart technology solutions need to be adapted to suit an ageing society and satisfy a high QoL [3,6]. Information and Communication Technologies (ICT) see a constant and rapid development, and provide a very powerful tool that can help in creating models and enable achievement of the ultimate goal, namely that of an age-friendly society with an improved QoL [7,8,9]. Yet, it is important that those ICT solutions are directed towards reaching the goal of personalised ageing and are initially designed and built to contribute to the QoL paradigm. 

According to the abovementioned, at the centre of the vision on personalised ageing is the older adult who aspires to live independently and in safety. A good ICT solution that reaches this vision has to perceive an older adult’s unique conditions and lifestyle. Therefore, the development of such an ICT solution should respect the older adult’s way of living, so that one can perform daily routines undisturbed by the technology that contributes to one’s QoL. 

This also means that ICT should serve the need of the end-users, instead of being fancy gadgets with a finite lifespan and a limited applicability. Therefore, efforts and research activities should be directed towards making useful models and effective technologies that contribute to QoL and personalised ageing. This forms the foundation of the first of the three aims of this paper: the proposal of a framework for the QoL for older adults through a systematic review of relevant state-of-the-art articles and patents, i.e., personalised ageing which can further be utilised by various stakeholders on this path. 

However, in order to utilise the future ICT solutions as parts of a smart environment that helps achieve personalised ageing with an increased QoL, one must first determine whether the existing ICT solutions are satisfying the needs of older people. In other words, are these solutions successful in their performance. The existing research shows that current ICT solutions are partly succeeding in the direct improvement of the QoL of older people [10]. Therefore, the second aim of this paper is to investigate to what extent ICT solutions directly improve QoL for older people by surveying the wider scope of research papers from the relevant databases and patents. The third aim is to draw conclusions and guidelines for future ICT solutions for older adults, so that we could optimise their implementation. 

This paper is organised as follows: Section 2 describes the notions of smart ageing, QoL, and personalised ageing and their mutual interplay. Moreover, it presents a framework for the quality of life of older adults that shaped this review. Section 3 provides a detailed review methodology: data sources and search strategy, study selection processes (inclusion and exclusion criteria), and publications review and data abstraction. In other words, this part of the paper describes used methodology and provides meta-analysis. Section 4 gives the results of the conducted survey, while Section 5 discusses the obtained results, recognises the gaps and opens research questions for future studies, and describes study limitations. Finally, Section 6 provides the conclusions of the paper. 

## 2. The Framework for Quality of Life and Personalised Ageing

This section introduces and elaborates the terms of quality of life and smart ageing, and discusses ICT solutions as building blocks that sync into the concept of personalised ageing, i.e., achieving quality of life for older adults. It provides the description of the proposed framework for achieving an increased QoL for older adults, i.e., personalised ageing, from the perspective of ICT solutions. This proposed framework is used for the structured review of the literature and patents, and serves as a basis for the presentation of the results of both reviews.

### 2.1. Quality of Life

Quality of life is a broad concept that has many definitions and meanings depending on the context under consideration. It is referred to as [11]: “a perception of one’s position in life in the context of culture and value systems in which they live and in relation to their goals, expectations, standards, and concerns”. The QoL is culturally built as a person’s search to satisfy the three universal requirements, i.e., (1) safety and security, (2) a sense of integrity and meaning of life, and (3) a sense of belonging to a social network. As will be discussed below, this means that culture contributes to three QoL dimensions (i.e., economic and physical safety; overall experience of life; and leisure and social interactions). It is also specified in terms of well-being [12]: “a state of well-being which is a composite of two components: (1) the ability to perform everyday activities which reflect physical, psychological, and social well-being, and (2) patient satisfaction with levels of functioning and the control of disease and/or treatment related symptoms”. Others use the term satisfaction with life [13]: “QoL is the degree of need and satisfaction within the physical, psychological, social, activity, material, and structural area”. One can conclude that QoL is something like the overall enjoyment of life and a multidimensional concept which emphasises the self-perceptions of an individual’s current state of mind, which is affected in a complex way by the person’s physical health, psychological state, personal beliefs, social relationships, and their relationship to salient features of their environment [14].

According to the European Framework 8 + 1 [15], QoL constitutes the following eight dimensions: (i) material living conditions; (ii) health; (iii) education; (iv) productive and valued activities; (v) governance and basic rights; (vi) leisure and social interactions; (vii) natural and living environment; (viii) economic and physical safety; and lastly (ix) overall experience of life. Material living conditions cover the income, consumption, and material conditions of a person. Productive and valued activities relate to economic activity, i.e., quality and quantity of employment, as well as other activities, i.e., inactivity and unpaid work. Health is measured in outcomes, healthy and unhealthy behaviours, and access to healthcare. Education covers competences and skills, lifelong learning, and opportunities for education. Leisure and social interactions include quantity and quality of leisure, as well as access to leisure, and the social dimension, i.e., relations with people and activities for people together with social support and cohesion. Economic security and physical safety address wealth (assets), debt, and income insecurity from the economic side, and crime and a perception of physical safety from the physical side. Governance and basic rights cover institutions and public services, discrimination and equal opportunities, and active citizenship. Natural and living environments include pollution, access to green and recreation spaces, as well as landscape and built environments. The ninth dimension, i.e., overall experience of life, covers life satisfaction, effects (negative—being nervous or being depressed or down, and positive—being happy), and meaning and purpose of life.

### 2.2. Smart Ageing

Smart ageing is a term that is often used interchangeably with healthy ageing [16] or active ageing [17], but without an exact definition. One description explains it as a wide concept defined as technology and innovation usage in both the public and private sectors to produce products, services, solutions, and systems to improve the QoL of people who are 50 years old and over [18]. A similar definition is given by Varnai et al. in [19] and is as follows: smart ageing is using technology, innovation and design in both the public and private sectors to produce products, services, solutions, and systems to improve the quality of life for the older generation in three key areas: functional food, connected health, and assisted living. Moreover, Song et al. in [20] regards smart ageing as a concept for mitigating the effects of ageing and improving older peoples’ life by managing various healthcare challenges with the utilisation of biomedical, computing, and communication technologies. 

According to EuroHealthNet [16], the key determinants of smart ageing are: (i) access to services; (ii) employment and volunteering; (iii) physical activity; (iv) social inclusion and participation; (v) new technologies; (vi) diet and nutrition; (vii) long-term care; (viii) environment and accessibility; and (ix) education and life-long learning. Namely, appropriate health and social services should be accessible to all older people. It is important that older people are not excluded by a new means of delivering services (like a shift from personal care to online services). Being employed or involved in voluntary activities is a great way of maintaining health and avoiding social exclusion. In adults aged 65 years and over, physical activity includes leisure time; physical activity; transportation; occupations; household chores; play; games; sports; or planned exercise, in the context of daily, family, and community activities [21]. Social inclusion and participation in various activities enables older people to grasp opportunities to be active and breaks down barriers they face in their everyday lives, such as cognitive impairment and depression [22], a lack of stimulation and social interaction [23], lethargy, boredom, depression, and loneliness [24]. New technologies are developing at a rapid pace and affect every segment of our lives. ICT can and will play a key part in helping older people to be more independent and to lead healthier lives. In this regard, many ICT solutions are proposed to prolong and support the independent and active living of older adults. Therefore, those solutions contribute to long-term care by providing possibilities to monitor the activities and health of older adults. ICT solutions for activities monitoring are deployed at home to warn caregivers about any unusual behaviour in older adults or outside the home to control risky situations. On the other hand, ICT solutions for health monitoring combine ICT solutions for activity monitoring at home with the use of medical devices. However, those solutions have to address several challenges and barriers, i.e., (i) ease-of-use because many older adults are not comfortable with technologies; (ii) invisibility and disuse to isolate older adults; (iii) privacy and security to avoid older adults becoming vulnerable considering their health conditions; (iv) affordability of technology in terms of cost; and (v) supporting older people to stay in their homes or move in different environments independently. Ageing affects nutrition as well as lifestyle: adequate nutrition becomes increasingly difficult with increasing age, whereas physical activity usually decreases. Nutrition and lifestyle, however, are important determinants of health and outcome in older people, especially in those with multiple chronic conditions [25]. Nutritional status, dietary habits, and food patterns vary widely across Europe [26]. Moreover, the quality and accessibility of the environment in which an older person lives can have a significant bearing on how active they are in society. Finally, there is a strong connection between learning and better health for older people. The concept of life-long learning does not mean only obtaining employment-related qualifications, it also means promoting learning throughout the life course, for the well-being and enjoyment of all. 

### 2.3. The Framework—A Contribution of ICT Solutions

**Figure 1:**
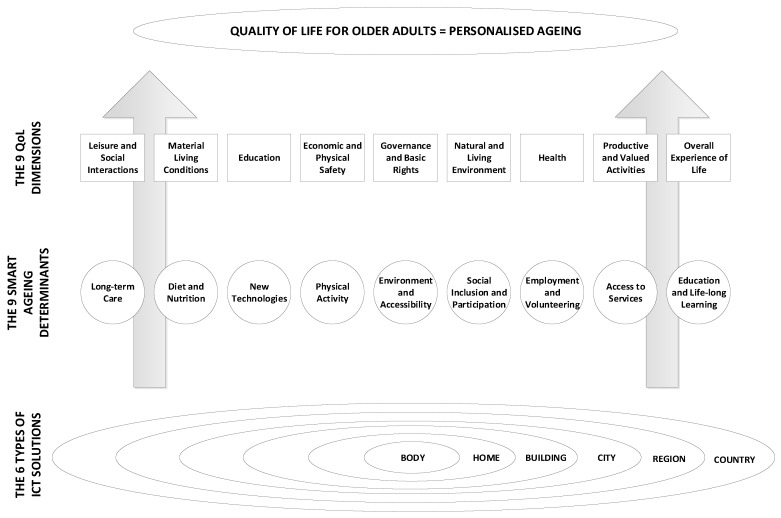
The framework for the Quality of Life (QoL) of older adults.

The elaboration on quality of life and smart ageing provide the necessary building blocks to introduce the framework of personalised ageing. Figure 1 represents our framework for achieving personalised ageing, i.e., the QoL of older adults. The proposed framework has a horizontal and a vertical component. 

#### 2.3.1. Horizontal Component

The horizontal component refers to and describes the environment in which the multiple ICT solutions will operate, further contributing to the smart ageing concept. As given in the framework, those can be the: (i) human body; (ii) home; (iii) building; (iv) city; (v) region; and (vi) country. Smart ICT body-worn solutions cover devices with installed tools, applications, or systems that will be placed on the human body, i.e., watches, glasses, bracelets, chips, and pacemakers. Smart ICT home solutions cover items that surround the older persons in their homes or narrow living environments such as smart furniture, phones, computers, spatial, and temporal sensors. Both types of solutions should aid older adults in their more confident and independent behaviour in the domestic environment. Furthermore, these solutions could be totally personalised to older individuals. Smart ICT building solutions include the rule of operation, and devices that older persons encounter and use as soon as they close their home doors, but before they are out in the streets and concern things such as stairs, elevators, keys, etc. Smart ICT urban solutions refer to solutions (logic, applications, systems, devices) that are placed anywhere in the city (or a village) and increase the ease the life of older adults when they come across environments that include public transport and public surfaces, various spatial and temporal sensors, and so on. ICT building solutions and city/urban solutions are ones that are used by other cohorts in the population too, but they need to be adjustable to older adults considering the fact that they will soon make up a quarter of the entire world population. Moreover, these solutions should encourage and support older adults to take their deserved place in society instead of withdrawing from it. Smart ICT regional solutions cover applications and systems that provide better governance of certain regions taking into account their specificities, while smart ICT country solutions refer to those that affect the country as a whole, and are mostly regulatory and policy related. The latter two also affect the other age groups in society, but they should be conceptualised in an adjustable fashion in order to satisfy the needs of older adults. 

#### 2.3.2. Vertical Component 

Taking the bottom-top approach, the description of the vertical component of the framework aimed at the QoL of older adults is as follows: The first step on our way to a better QoL are ICT solutions (Figure 1). That is, the existing ICT solutions and ones that are to be developed in the (near) future should be designed to contribute to key smart ageing determinants.

**Figure 2:**
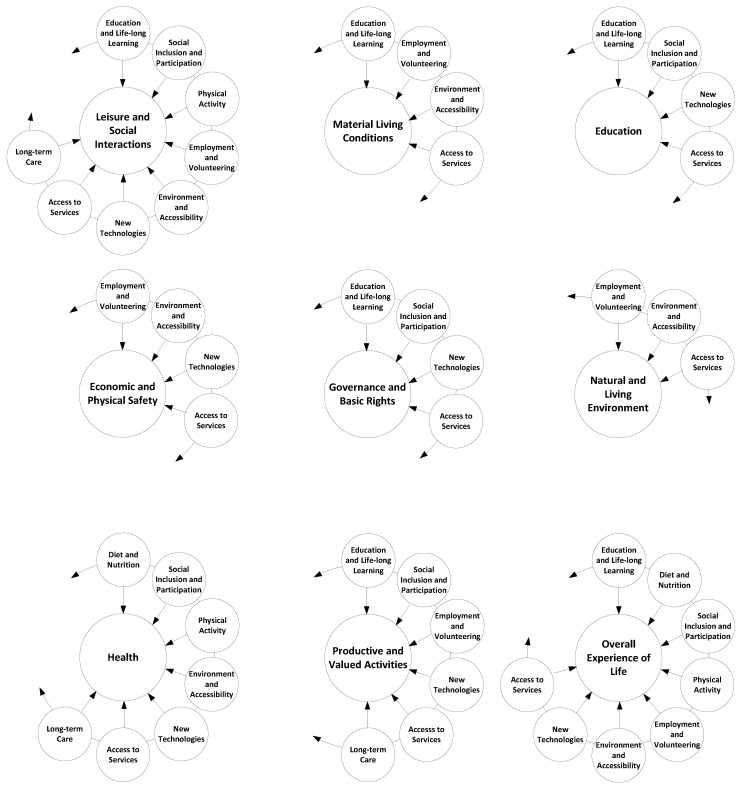
The proposed mapping of the smart determinants to QoL dimensions.

The next level is having smart ageing determinants mapped out into QoL dimensions. A proposition for the mapping is given in Figure 2. The main aim of this mapping is to have a link between these two concepts. The knowledge of these connections, i.e., which smart ageing determinant influences which QoL dimension, gives better targeting opportunities and effective attainment of the ultimate goal—QoL for older adults, i.e., personalised ageing. The main aim of this mapping is to find a link between these two concepts (i.e., smart ageing determinants and QoL dimensions). For example, an ICT tool that is designed to satisfy the smart ageing determinant education and life-long learning can positively affect and improve the QoL for older adults by contributing to its following dimensions. First, leisure and social Interactions, because it allows older adults to entertain themselves and to get in touch with other learners. Second, material living conditions, because it allows older persons to increase their incomes by doing various paid jobs if they increase their knowledge in a specific domain. Third, education, because it allows them to have personal growth. Fourth, health, because it allows older adults to take better care of their physical and mental health if they constantly learn about the new medical improvements and health recommendations. Fifth, productive and valued activities, because it allows older adults to feel productive. Finally sixth, the overall experience of life, because it allows them to feel better if they enjoy learning.

On the other hand, QoL dimensions on material living conditions can be affected by the following smart ageing determinants: education and life-long learning; employment and volunteering; environment and accessibility; and access to services. For example, education and life-long learning allows older adults to gain knowledge they can monetise and improve their material status. Employment means that older adults can work and thereby improve their material living conditions. Environment and accessibility as well as access to services impact material living conditions as a QoL dimension in terms of providing opportunities to allow older adults to increase the level of their material living conditions.

It is also important to stress that in addition to the impact that these smart ageing determinants individually have on QoL dimensions, they can also have interdependent relations among each other. These interplays between them as well as constructs that they form which in the end can affect QoL dimensions should be additionally investigated in future research. 

The final level is formed by the QoL constituents (in this framework called QoL dimensions (8 + 1) [15]) a contribution to the improvement of the overall QoL of older adults, which leads to personalised ageing. In case there are multiple ICT solutions designed to be personalised and satisfy multiple smart ageing determinants, which in combination contribute to multiple QoL dimensions, then those improved QoL dimensions jointly accomplish the goal of QoL while ageing or in personalised ageing.

## 3. Methodology of the Review

The following paragraphs describe the methodology for the reviewing process, namely data sources and search strategy; the study selection process; the review of publications review and data extraction.

### 3.1. Data Sources and Search Strategy

As research on smart ageing and ICT solutions for QoL has been conducted in multiple scientific domains, and scientific findings have been published in different literature repositories, our search strategy focused on two data sources, i.e., scientific articles and intellectual property (IP) patents. 

The article search was undertaken from January 2019 to March 2019 in order to identify published peer-reviewed articles in English. The databases searched included Web of Science (WoS), IEEE Xplore, and Scopus (the first source from 1999 until the last in 2019). The papers were extracted from databases using the following search phrases alone or in different combinations using logical operators of “AND” and “OR”: smart elderly, smart aging, ambient assisted living, and ambient assistance living.

The patents search was performed in the ESPACENET database. The ESPACENET database was searched from November 2019 to March 2020 with the aim of finding relevant patents. The database search included the following keywords in “topic” search (title or abstract or claims): (smart AND elderly) OR (smart AND aging AND living AND elderly) OR (smart AND aging AND ambient AND assisted AND living AND elderly) OR (ambient assisted living) OR (ambient assistance living).

### 3.2. Study Selection Process

**Figure 3:**
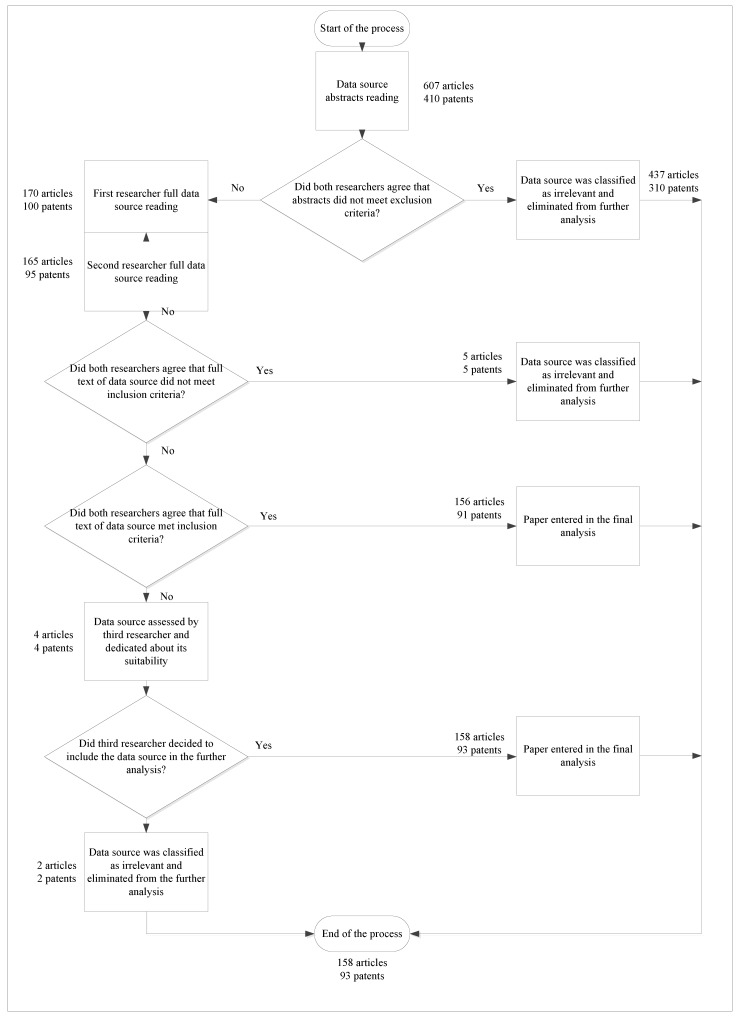
The data source selection process.

The titles and abstracts retrieved by the database search were analysed in order to select data sources (i.e., articles and patents) that satisfied the inclusion criteria. Two independent researchers (ZA and SB for articles, SB and JBH for patents) evaluated the titles and abstracts and compared them to the inclusion and exclusion criteria. They met in order to reach a consensus through discussion. In case of different opinions regarding the abstract’s suitability, the given data source was included for further analysis. Further on, the two researchers surveyed the full texts of the selected data sources. In order to determine suitability for inclusion in further analysis, one researcher assessed all the articles and the other one evaluated all the patents. After data abstraction of the final selected data sources (i.e., articles and patents), two new researchers independently surveyed 20% of randomly selected articles and patents. If there was any disagreement on the suitability of data sources, advice was asked from a third researcher (JBH for articles, OK for patents) for an evaluation of the given data source. A third researcher was an interdisciplinary expert that provided a final decision whether or not to include the data source in the analysis. Figure 3 (inspired by [27]) shows the data source selection process in more detail.

#### 3.2.1. Inclusion Criteria

The inclusion criteria were as follows:

- Articles and patents which included ICT solutions to achieve the QoL for older adults that:
  1. Addressed ICT solutions in home or supportive care environments for older adults with specific needs regardless of whether the ICT solution was embedded on the human body or in a home, building, city, region, and country;
  2. Addressed the physical and/or mental needs faced by older adults;
  3. Involved ICT solutions that have been implemented or deployed in pilot form contributing to the key smart ageing determinants (i.e., long-term care, diet and nutrition, new technologies, physical activity, social inclusion and participation, employment and volunteering, access to services, and education and life-long learning);
  4. Articles and patents which intend to understand various QoL dimensions (i.e., material living conditions, health, education, productive and valued activities, governance and basic rights, leisure and social interactions, natural and living environment, economic and physical safety, and overall experience of life) in terms of QoL for older adults;
  5. Articles and patents which are peer-reviewed and published in English within a 20-year period (i.e., 1999–2019).
  6. Articles that used any kind of research methodology with positive/negative results.

#### 3.2.2. Exclusion Criteria

The exclusion criteria were as follows:

- Articles and patents written in a language other than English;
- Articles and patents that were unavailable or available with an abstract;
- Articles covering theoretical reviews, narrative reviews, meta-analyses, conference notes, and other types of literature reviews;
- Articles and patents that are about technology which differs from ICT for older people;
- Research published in conference proceedings, books, book chapters, and master’s and doctoral theses;
- Articles and patents that consider ICT solutions contributing to smart concepts other than smart ageing and QoL for older adults;
- Articles and patents on assistive devices such as canes, wheeled walkers, hearing aids, etc.;
- Studies published before 1999;
- Research which focuses on ICT solutions contributing to the concept of smart ageing in developing countries, which are under different conditions to developed countries;
- Research with a focus on industry and production of ICT solutions;
- Research materials on telemonitoring, telemedicine or telehealth programs which include self-monitoring using low-complexity technologies;
- Research materials that reported combined interventions;
- Articles that did not provide enough information for categorising the data source.

### 3.3. Publications Review and Data Extraction

The analysis was conducted by an inductive approach. The results section was divided into two parts, i.e., an article analysis and a patent analysis. A detailed summary of each article and patent was provided. Throughout this process, the following items were systematically extracted: authors, titles, publication place, database (WoS, Scopus, Xplore), QoL dimensions (previously listed and described in Section 2.1), smart ageing determinants (previously listed and described in Section 2.2), service users (older adults, care members, family members, other), validation indication (has the solution been validated by the older adults?), and operating environment (body, home, building, city, region, and country). Then, the extracted fields were organised according to the framework in order to provide a structured overview of ICT studies contributing to QoL for older adults, i.e., personalised ageing. In addition, we used those fields to perform the meta-analysis of both selected articles and patents. The meta-analysis enabled the quantification of articles (in Section 4.1) and patents (in Section 4.2), which include ICT solutions to achieve the QoL for older adults in order to provide the input data for discussion and conclusions. 

## 4. Results

In the following sections, the results of both the literature analysis and the patent analysis are described in more detail.

### 4.1. Literature Analysis

A total of 607 articles were identified during the initial search phase. After removing duplicates and applying the inclusion and exclusion criteria, only 158 studies were included in the final meta-analysis and data-abstraction phase (26%). Approximately 86% of them were found through the WoS database, while in Scopus and Xplore databases 11.5% and 2.5% of studies were found. It is important to note that the selected papers that are in WoS but also in other subject databases are categorised as WoS papers, while the ones that were only in Scopus or Xplore are marked accordingly. When it comes to the distribution by year, a total of 4.43% of articles were published between 1999 to 2009, and 31% between 2010 to 2014. Articles published in the period from 2015 to 2019 count for 12%, 14%, 18.4%, 16.5%, and 3.6% for each year respectively (Supplementary Materials).

Taking the bottom-up approach, the operational environment was addressed firstly (in accordance with Figure 1). The obtained results show that currently the most represented solutions are the ones for the home environment (66%, i.e., 104 articles out of 158 studies), followed by the ones that are related to the human body (24%, i.e., 38 articles out of 158 studies). Solutions categorised as building, city, region, and country count for a mere 1%, 4%, 0%, and 5%, respectively.

Further on, the addressed ICT solutions that are designed for smart ageing contribute to 7 out of 9 smart ageing determinants, which are represented in the following percentages: 36% for physical activity, 18% for new technologies, 16% for long-term care, 14% each for environment and accessibility and social inclusion and participation, and 1% each for diet and nutrition and access to services. Missing determinants are employment and volunteering and education and life-long learning. Moreover, the currently represented smart ageing determinants can be mapped to 7 out of 9 QoL dimensions, i.e., health (30%), overall experience of life (23%), economic and physical safety (17%), material living conditions (13%), natural and living environment (9%), leisure and social interactions (7%), and productive and values activities (1%). Missing QoL dimensions are education and governance and basic rights. 

When it comes to the mapping of addressed smart ageing determinants to the addressed QoL dimensions, in accordance to the obtained results, the leisure and social interaction QoL dimension is impacted by ICT solutions designed to satisfy the following smart determinants: social inclusion and participation (7 studies), physical activity (2 studies), and environment and accessibility (1 study). Material and living conditions have been affected by ICT solutions for physical activity (7 studies), new technologies (6 studies), environment and accessibility (5 studies), and long-term care (3 studies). ICT solutions designed for physical activity (16 studies), long-term care (6 studies), environment and accessibility (2 studies), access to services, new technologies, social inclusion, and participation (1 study each) contribute to economic and physical safety. The natural and living environment is influenced by the ICT solutions for the next smart ageing determinants: physical activity (7 studies), environment and accessibility (5 studies), and long-term care and new technologies (1 study each). Physical activity related to ICT solutions (2 studies) are mapped to productive and valued activities as QoL dimensions. The QoL dimensions that the most diverse ICT solutions are mapped to are health and overall experience of life. Physical activity and long-term care (15 studies each), new technologies (10 studies), environment and accessibility (3 studies), social inclusion and participation (2 studies), and access to services; diet and nutrition; education and lifelong learning (1 study each) related to ICT solutions are mapped to health, while new technologies (12 studies), physical activity (9 studies), environment and accessibility (7 studies), social inclusion and participation (3 studies), and access to services; diet and nutrition; and long-term care (2 studies each) ICT solutions are mapped to the QoL dimension and overall experience of life.

Additional items that were investigated in this study were whether the addressed selected studies and the ICT solutions that they propose are made for older adults or for the care of a family member or other, and whether these solutions were validated by the aimed end users. Obtained results show that 60.8% of studies propose solutions for older adults, 24.7% for care members, 13.9% for others (such as scientists, data collectors, etc.), and only one study was exclusively for family members. We have found that 78.5% of the considered solutions are validated by the targeted group of end-users, while the remaining 21.5% are not.

### 4.2. Patent Analysis

A total of 410 patents were identified during the initial search phase. After removing duplicates and applying the inclusion and exclusion criteria, only 93 patents were included in the final meta-analysis and data-abstraction phase (22.7%). All of the patents were taken from ESPACNET. When it comes to the distribution by year, we found no relevant patents in the period 1999–2009. In the period 2010–2019, a total of 13% of the patents were published between 2010 and 2014, and patents published between 2015 and 2019 count for 3.2%, 6.4%, 14%, 32.2%, and 31.2% for each year respectively. 

Applying the bottom-up approach again, the operational environment was firstly addressed. The obtained results show that currently, the most represented solutions covered by patents are the ones for the home environment (45.1%, i.e., 42 out of 93 patents). Second place is taken by the ICT solutions related to the human body (26.9%, i.e., 25 out of 93 patents). ICT solutions categorised as building, city, region, and country count for 3.2%, 0%, 1%, and 23.8%, respectively.

The addressed ICT solutions in patents designed for smart ageing contribute to 7 out of 9 smart ageing determinants. This is the same as for the literature review. Missing determinants are employment and volunteering and education and life-long learning as it was the case with the literature as well. The percentages are: long-term care with 50.5%; new technologies with 28%; physical activity with 7.5%; social inclusion and participation with 4.3%; and access to services, diet and nutrition, and environment and accessibility with 3.2% each. The currently represented smart ageing determinants in the patent analysis can be mapped to 6 out of 9 QoL dimensions, i.e., health (65.6%); leisure, social interactions, and economic and physical safety (8.6%); overall experience of life (7.5%); material living conditions (5.4%); and natural and living environment (4.3%). The missing QoL dimensions are education and governance and basic rights with added productive and valued activities. This corresponds to the outcomes of the literature review.

Regarding the mapping of the addressed smart ageing determinants to addressed QoL dimensions in patents analysis, in accordance to the obtained results, leisure and social interaction QoL dimensions are impacted by ICT solutions designed to satisfy the following smart determinants: social inclusion and participation (4 items); new technologies (2 items); and access to services (1 item). Material and living conditions have been affected by ICT solutions for new technologies (4 items) and physical activity (1 item). ICT solutions designed for new technologies (4 items), long-term care (2 items), and physical activity and access to services (1 item each) contribute to economic and physical safety. Natural and living environment is influenced by the ICT solutions for the following smart ageing determinants: environment and accessibility (3 items) and long-term care (1 item). Overall experience of life is covered by ICT solutions contributing to new technologies (6 items) and long-term care (1 item) smart determinants. The QoL dimension that the most diverse ICT solutions are mapped to is health. Long-term care (43 items), new technologies (9 items), physical activity (5 items), diet and nutrition (3 items), and access to services (1 item) related to ICT solutions are mapped to health.

An additional question that was addressed in this study is whether the addressed selected patents and the proposed ICT solutions are made for older adults or for carers or relatives or other persons. The obtained results show that 88% of patents propose solutions for the older adults (44%) and carers (44%), while 12% propose solutions for relatives. No evidence was found that the selected patents were validated by the targeted end-user groups.

**Figure 4:**
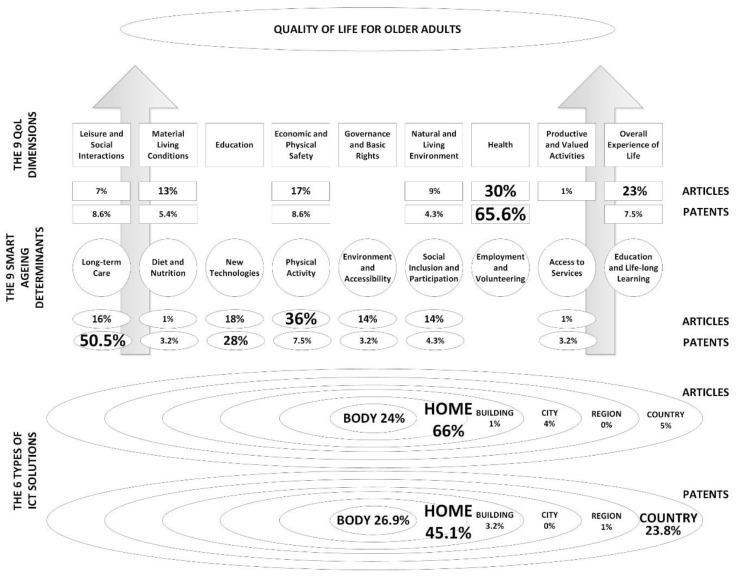
Summary of joint literature and patent analysis results in accordance with the framework on Figure 1.

Finally, the summary of joint literature and patent analysis results according to Figure 1 is given in Figure 4.

## 5. Discussion

Results discussion is organised related to proposed framework consisting of horizontal and vertical components. From the horizontal component perspective, the proposed framework for achieving an improved QoL for older results and personalised ageing calls for a balanced distribution of ICT solutions across different application places such as body, home, city, building, region, and country (Figure 1). If the solutions are represented in a balanced manner, they create a basis for personalised ageing in combination. From the vertical component perspective, the framework advocates having ICT solutions which are designed to satisfy all nine different smart ageing determinants (Figure 1). This does not imply that one should have ICT solutions that simultaneously contribute to all nine determinants, but to one or several different ones if possible. Moreover, the framework proposes adequate mapping of smart ageing determinants to nine different QoL dimensions which ultimately would result in improved overall QoL for older adults (Figure 1 and Figure 2). The following subsections elaborate these findings based on horizontal and vertical components of the proposed framework in more detail, as well as study flaws and limitations.

### 5.1. Horizontal Component 

The obtained results of this structured review deviate from the horizontal component of the framework in several segments. Firstly, the article analysis shows that 90% of the ICT solutions are home- and body-focused, while the patent analysis shows that a lower percentage of patents of 72 is focused on such solutions. Very often, this is in connection with the monitoring of movement, certain body functions, and the spaces where a person moves around (bedroom, bathroom, and other selected parts of the apartment). Another finding of this study is that many ICT solutions can be identified in the field of motion tracking and monitoring of older people, in particular, in the domain of fall detection, tracking, and taking medication. When implementing these complex solutions, people run into the limits of the health care system’s readiness to process the large amount of data that becomes available and can be shared. 

The vast majority of existing body solutions are focused on solving a partial problem, as well as complex solutions where a home control unit is provided. Such home control units can be connected to, and interface with, a combination of health equipment, smart home appliances, smart medicine cabinets, a smart pantry, wearable sensors, motion detectors, video cameras, microphones, video monitors, speakers, smart thermostats, lighting, floor sensors, bed sensors, smoke detectors, glass breakage detectors, door sensors, and other perimeter sensors [28]. Similar findings are confirmed by other studies. For example, Haghi et al. [29] reviewed wearable health care devices, both in the literature as in commercial efforts. Their study showed that a vast array of wearables, with the help of improved technology, are considered to be reliable tools for long-term health monitoring systems. Furthermore, Yin et al. [30] showed that the rapidly advancing information technologies and the emerging Internet of Things (IoT) technology have provided great opportunities for developing smart healthcare information systems. Nevertheless, challenges still exist in achieving secure and effective telecare applications. In their view, identified areas for future improvement are listed as follows: self-learning and self-improvement; hardware; and standardisation or privacy and security. In terms of hardware, the question of how to achieve unobtrusiveness still poses a big challenge, as comfort is naturally a key concern. Actually, the need to integrate multiple sensors into one solution contradicts the goal of unobtrusiveness. Furthermore, a number of research teams and organisations have contributed to the deployment and standardisation of IoT technologies. Finally, the two main prerequisites of applying IoT-based systems are their utility and safety for users. The privacy of older adults must be ensured in order to prevent unauthorised identification and tracking. From this perspective, the higher the level of autonomy and intelligence of IoT items, the more challenges concerning the protection of identities and privacy would come to the fore [31,32]. Oude Weernink et al. [33] further distinguish between the settings in which such technologies are used, for instance, the nursing home or hospital environment versus the own home or on the street, and whether technologies are used to track or monitor older people, or even their belongings, as part of a maintenance protocol. In conclusion, a proper analysis of the goals, settings, and potential users (and their knowledge and awareness concerning technology, for instance, in the case of dementia) of technologies needs to be made before introducing technologies into the daily lives of older people.

Although the contribution of the selected ICT solutions to personalised ageing and improving QoL for older adults in body and home domains is not questionable, there seems to be a gap. What is currently missing from a more comprehensive approach to the topics of personalised ageing and QoL is a greater focus on urban-related ICT solutions, i.e., buildings, cities, regions, countries. The society as a whole should not consider older adults as individuals whose place is in their homes and controlled environments only. Ideally, older people should be given better conditions to cohabitate with younger representatives of society in all places worldwide and contribute accordingly. Therefore, the gap that should be covered by future research is the investigation and creation of ICT solutions for urban ageing, i.e., related to buildings, cities, regions, and countries. This recommendation further adds to the agenda of the WHO Age-friendly Cities and Communities programme [7].

### 5.2. Vertical Component 

The existing ICT solutions deviate from the horizontal component of the framework in terms of a balanced contribution to different smart ageing determinants. The analysis of the literature shows the dominance of physical activity-related ICT solutions with a minor representation of solutions contributing to long-term care, new technologies, the environment and accessibility, and social inclusion and participation. The patent analysis is dominated by ICT solutions contributing to long-term care and new technologies. The lack of a balanced distribution of ICT solutions across all smart ageing determinants leads to a lack of a multidimensional contribution to different QoL dimensions, and ultimately, an improved overall QoL for older adults and personalised ageing. A second recommendation to be addressed in future investigation is the creation of a balanced representation of ICT solutions across different smart ageing determinants. For instance, there is a need for more work on ICT solutions focused on the underrepresented smart ageing determinants such as access to services, employment and volunteering, education, and life-long learning to name a few.

One can also conclude the same when it comes to the representation of the addressed QoL dimensions in the reviewed literature and patents. Current research and ICT solutions, as well as review studies, are mostly focused on health, overall experienced QoL, or economic or physical safety. Improved QoL, in general, is not improved by enhancing only these three dimensions, but all of them in interdependence of the personal preferences of an individual. Therefore, if one wants to have personalised ageing which assumes an improved QoL, it is suggested that the future research work provides ICT solutions covering a variety of QoL dimensions.

**Figure 5:**
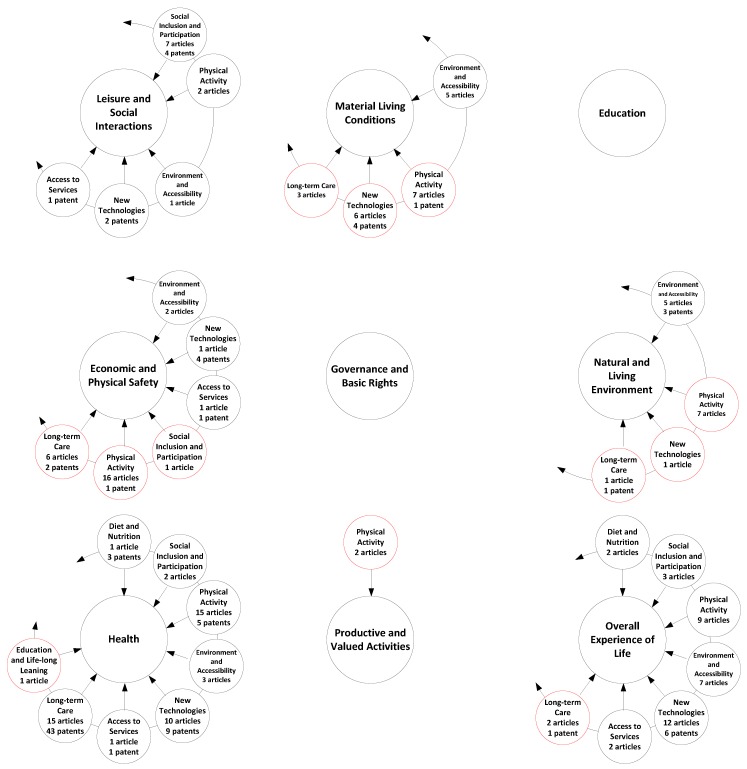
A superimposition of the results of the literature and patent review in terms of smart determinants to QoL dimensions show the gaps where scientific and R&D focus can be placed in the future, and where there is still room for scientific findings to be translated into potential patents. Newly added circles in red and omitted circles from the circle spoke diagram denote deviations from the proposed mapping.

The mapping of smart ageing determinants to QoL dimensions that is in line with the proposed framework is another topic for discussion. The analysed literature has a better performance in terms of mapping in comparison to the reviewed patents. For example, our framework suggests enhancing the QoL dimension “overall experience of life” by eight different smart ageing determinants (Figure 2). In the literature, the overall experience of life is impacted by ICT solutions belonging to seven different smart ageing determinants (such as new technologies, physical activity, environment and accessibility, and so on), while in patents this dimension is influenced only by ICT solutions addressing new technologies and long-term care as smart ageing determinants (Figure 5). Although articles show a more innovative approach to improving QoL dimensions and overall QoL for older adults by influencing these domains by ICT solutions stemming from different smart ageing determinants, this approach needs major improvement. Future research activities should focus on ICT solutions that improve the QoL dimensions indirectly through a contribution to different smart ageing determinants.

It is important to note that it is hard to compare this part of the results, which are related to the proposed framework (the first aim of the study), to other studies given that the existing reviews and studies that have proposed frameworks [33,34,35,36,37,38,39,40] have not addressed personalised ageing and QoL improvement for older adults through the prism of ICT solutions. Many ICT solutions are proposed with the target to prolong and support the independent living of older adults and provide help for professional and informal carers [41]. In order to build an ecosystem that could satisfy the needs of carers, these ICT solutions should provide older adults with control over the timing and the place for monitoring their health [27], while at the same time reducing the stress on hospital capacity and care institutions [42,43]. However, there is a lack of standards, safety and interoperability of these ICT solutions, as well as methods for validation and verification methods in order to demonstrate the sustainability and reliability of ICT solutions for older adults [34]. Therefore, the awareness of the potential ICT solutions for personalised ageing among different stakeholders should be increased. 

The second aim of this paper was to investigate to what extent ICT solutions do directly improve QoL for older people by surveying a wider scope of the literature and patents. Both analyses show that the majority of ICT solutions are intended for older adults and that they are validated by them. As already indicated, this is opposite to the results obtained by Baraković Husić et al. [10]. The reason for opposite results is the variation in the selected articles and patents, i.e., ICT solutions. Therefore, depending on the reviewed items, the answer to the question of whether solutions are intended for older adults can be different. However, it is recommended for future research to continue to focus more on personalised ICT solutions that directly improve the QoL of older people and to provide older people with the means to enjoy the highest level of independent participation in everyday activities regardless of the place. In order to achieve this goal, the research community should focus on understanding the needs of older people in order to create scalable and flexible ICT solutions that could be adjusted in accordance with personal preferences. It is, therefore, very important to embed the commitment to create ICT solutions in line with the personal needs of older individuals into future research procedures. 

### 5.3. Study Flaws and Limitations

Apart from the chosen application places represented in the framework for this study, there are additional application domains that could be considered, and which are derived from the taxonomy for gerontechnology by van Bronswijk et al. [44]. Gerontechnology aims at good health, full social participation, and independent living up to a high age, be it through research or the development or design of products and services to increase the QoL. According to the taxonomy, gerontechnology has five domains of application coupled with four types of technology impact. The application domains of the gerontechnology taxonomy include (i) health and self-esteem (“autonomy”); (ii) housing and daily living; (iii) mobility and transport; (iv) communication and governance; and (v) work and leisure. Technology impacts include (i) enhancement and satisfaction; (ii) prevention and engagement; (iii) compensation and assistance; and (iv) care support and organization [17]. These potential technology impacts especially could be investigated in more detail in future studies on ICT solutions and the improvement of QoL for older people. Contemporary examples of how technology can serve different communities and have various impacts can be found from the Technology In Later Life (TILL) study. This international, multi-centered, multi-methods study investigated how various technologies are used and impact on the leisure [45] and day-to-day activities of older adults living in both rural and urban geographic areas in the UK and Canada. Findings present two overarching themes: facilitators of technology use and detractors of technology; and numerous recommendations are proposed to move the existing debates forward in the area of gerontechnology and to reduce the notion of reinventing the wheel [46]. Age-friendly ecosystems relate to all citizens in society, and this includes dependent adults, careers, and children diagnosed with disabilities. Given the numerous technologies available at present and used by citizens, it is important to explore how such technologies can be used and deployed inside and outside of the home and across different communities in order to benefit the citizens in the respective age-friendly communities [47,48,49]. Whilst this framework was published by the World Health Organization in 2007 prior to significant technological developments, such technologies have been embraced by many citizens in their day-to-day lives and it illustrates the capabilities, forward thinking, and planning of existing and future ageing cohorts. There seems to be some implicit recognition of the role technology may play in realising the goals of the age-friendly cities and communities’ movement [34].

Our study suffers from several limitations as well. There is the possibility that some relevant publications are not included in the systematic review due to the specificity of the search strings. Moreover, some publications may have not been identified in this literature and patent review due to exclusion criteria (previously listed in Section 3.2.2). This especially refers to publications considering ICT solutions contributing to smart concepts other than smart ageing and QoL for older adults. In addition, this systematic review has not covered research with a focus on the industry and production of related ICT solutions. Furthermore, the considered publications were categorised by publication year and database, QoL dimensions, smart ageing determinants, operating environment, service users, and validation indication. This categorisation served to provide the quantitative results, while the qualitative results are covered to a limited extent. Finally, there were a considerable number of parameters that could not be examined because the publications in the domain did not report them. In order to have more accurate results, various aspects of older people’s QoL and personalised ageing need to be included in the equation of research activities.

## 6. Conclusions

As societies are ageing at a rapid speed, this research study aimed at contributing to the field of QoL for older adults and personalised ageing from an ICT perspective. The developments and the pace at which related solutions are brought to the marketplace, or are being implemented in the domain of healthcare and welfare, are ever-increasing. The main challenge is to put them into practice, thus taking a fundamental step towards improving the quality of life. This paper contributes to achieving this goal in three ways. 

The first contribution is the proposition of a novel framework for QoL for older adults through a systematic review of the relevant state-of-the-art articles and patents, i.e., personalised ageing which can be further utilised by various stakeholders on this path. Namely, the framework advocates the approach in which if we have multiple ICT solutions designed to be personalised and satisfy multiple smart ageing determinants which in combination contribute to multiple QoL dimensions, then those improved QoL dimensions jointly accomplish the goal of QoL while ageing or relating to personalised ageing.

Furthermore, in order to utilise the future ICT solutions, the current ones need to be investigated for satisfying the needs of older people. The second contribution is the finding that they are intended for older adults and are validated by them. However, this can vastly depend on the selected samples.

The third contribution of the study are the guidelines for future ICT solutions, so we could optimise their implementation. There are six recommendations that are derived from the review of the literature and the patents covering them: a focus on urban ageing, i.e., relating to buildings, cities, regions, and countries; the creation of a balanced representation across different smart ageing determinants; the cover of a variety of QoL dimensions; an improvement in the QoL dimensions indirectly through a contribution to different smart ageing determinants; an increase in the awareness of personalised ageing among different stakeholders; and to continue to directly improve the QoL of older people and to provide older people with the means to enjoy the highest level of independent participation in everyday activities regardless of the place.

## Supplementary Materials

The studies included in the literature analysis are available online at: https://drive.google.com/open?id=1qePpKiMK-4wkJzrm9b1FITRBtST17tuq. The patents included in the patent analysis are available online at https://drive.google.com/open?id=1qePpKiMK-4wkJzrm9b1FITRBtST17tuq.
